# Perforated mesenteric Meckel’s diverticulum: Case report

**DOI:** 10.1016/j.ijscr.2021.01.027

**Published:** 2021-01-15

**Authors:** Basem AlShareef, Orjuana Khudari

**Affiliations:** a Department Of General Surgery, College Of Medicine, Umm Al-Qura University, Makkah, Saudi Arabia; b Al-Noor Specialist Hospital, Department of General Surgery, Makkah, Saudi Arabia

**Keywords:** Perforated Meckel’s, Mesenteric Meckel’s diverticulum, Meckel’s case report

## Abstract

Meckel’s diverticulum is a common congenital anomaly found in ∼2% of the population, its classic location aiding in its diagnostic criteria, is the anti-mesenteric side of the distal ileum. However, reported cases of Meckel’s diverticula found on the mesenteric side, are present but rare. Here we report a case of a perforated mesenteric Meckel’s diverticulum with synchronised anti-mesenteric Meckel’s diverticulum, in a 70-year-old male, initially misdiagnosed as left strangulated inguinal hernia. The purpose behind this paper is to raise clinical suspicion regarding this rare pathology, so that timely diagnosis and management could be carried out.

## Highlights

• Meckel’s diverticulum has a variant in the mesenteric side.
• Mesenteric Meckel’s diverticula are often misdiagnosed until surgical intervention due to non-specific presentation.
• Complications are common presentation due to difficulty and delay in diagnosis.
• High clinical suspicion of Mesenteric Meckel’s diverticulum encouraged for acute abdomen.

## 1 Introduction

Meckel’s diverticulum is a remnant of the prenatal vitelline duct that failed to regresses between the fifth and seventh week of foetal life and is a common congenital anomaly found in ∼2% of the population. The anomaly’s clinical significance is that the persistent diverticulum can lead to complications such as intestinal obstruction, inflammation of the diverticulum, bleeding, ulceration or perforation, which may often be misdiagnosed. The anti-mesenteric side of the distal ileum, is the classic location of a Meckel’s diverticulum and aids in its diagnostic criteria. However, this diagnostic concept is challenged by few rare reported cases of Meckel’s diverticulum found on the mesenteric side of the ileum. Like previously reported cases, the pathology in our case was misdiagnosed at first; the pre-operative diagnosis made was strangulated inguinal hernia presenting with left groin swelling.

This work has been reported in line with the SCARE criteria [1].

## 2 Case report

70-year-old male patient, medically and surgically free, presented to the emergency department complaining of left groin swelling for 3 days’ duration. The patient’s swelling started gradually over a course of 3 days, associated with pain, nausea and vomiting, constipation and subjective fever. Patient denied previous history of trauma, chronic constipation and work-related heavy object lifting. Patient has no previous surgical history, no family history of similar presentation and no pharmacological history.

Physical examination: patient was conscious, alert and oriented; but hypotensive, tachycardic, tachypneic and in pain. Abdominal examination revealed a right sided inguinoscrotal swelling, around 20 × 15 cm, non-tender, reducible, with no skin changes. Also, there was another 5 × 7 cm left sided inguinal swelling but severely tender with bluish discoloration and hotness of the skin. Patient received initial fluid resuscitation, antibiotics and analgesia. Meanwhile, patient’s laboratory findings showed a white blood cell count of 27.55 × 10^9^/L, haemoglobin concentration of 8.1 g/dL, creatinine level of 484 μmol/L and urea level of 24.8 mmol/L. Electrolytes and other routine analysis were all within normal values. Patient’s chest and abdominal x-rays were unremarkable.

Patient was diagnosed with left strangulated inguinal hernia and was pushed directly to the operating room. Intraoperatively, a left inguinal approach was used. Around 20 cc of pure pus was drained, and the bowel looked healthy to the surgeon; so no resection was done and the hernia was repaired primary without mesh.

Post-operatively, the patient was doing fine, tolerating oral feeding and passing stool. On post-operative day 4, the patient became febrile with a temperature of 39 and his leukocytic count increased up to 30. Therefore, abdominal CT was done, which reported unremarkable findings, except for some subcutaneous oedema which was excepted post-operatively. Yet the patient was still sick.

On post-operative day 5, the wound was draining faecal matter. Patient underwent urgent exploratory laparotomy, where two Meckel’s diverticula at the terminal ileum were identified; one of them was perforated in the intra-abdominal wall, communicating with the left inguinal opening through the retro-pubic canal. Release of the bowel from the abdominal wall was done, resection of the part that contained the diverticulum was also done, with side to side anastomosis.

**Images 1 and 2:**
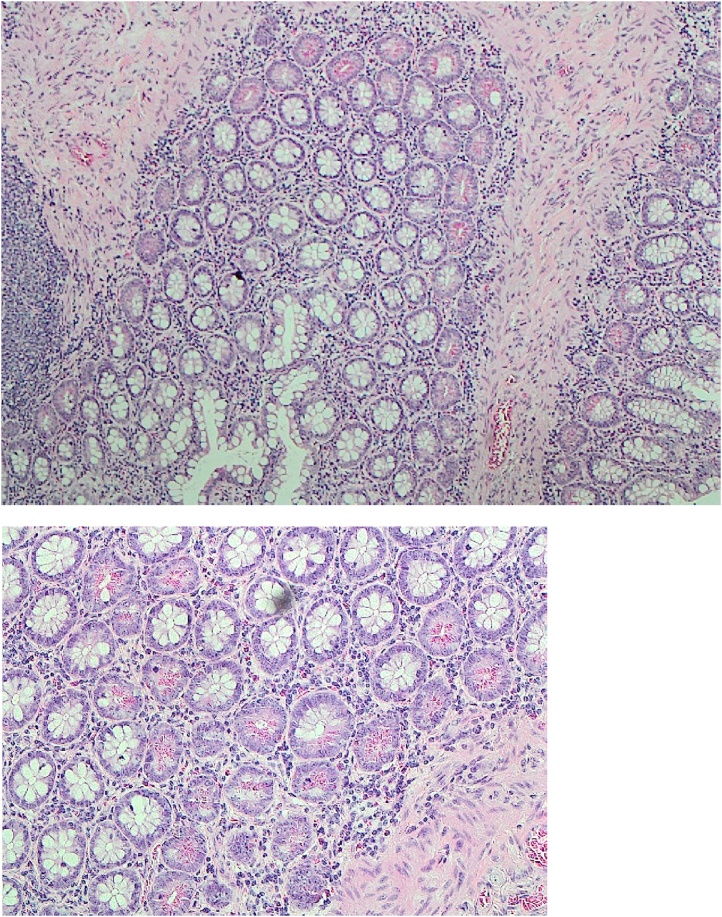
Histopathology images of our patient’s mesenteric Meckel’s diverticulum.

As for the two Meckel’s diverticula; one was on the anti-mesenteric side, while the other was on the mesenteric side, which was perforated. Histopathology result of taken samples revealed perforated Meckel’s diverticulum associated with dense inflammation and ischemia (Images 1 and 2).

Post operatively, the patient was kept in high dependency unit, NPO and on antibiotics. On post-operative day 4, patient spiked fever again with a sudden onset of severe lower abdominal pain. The patient underwent CT abdomen and pelvis which showed complete disruption of the anastomotic site. Another laparotomy was carried out were the anastomotic site was taken down and double barrel ileostomy was created.

The operator was a general surgery consultant with years of experience. In the following post-operative period, the patient unfortunately acquired a pulmonary embolism and passed away and no further follow up nor outcomes could be measured.

## 3 Discussion

Meckel’s diverticulum, known as the most common malformation of the gastrointestinal tract, is a remnant of the prenatal vitelline duct that failed to regress between the fifth and seventh week of foetal life, first described by Johann Friedrick Meckel in 1809 [1].

The diagnostic criteria for Meckel’s diverticulum include the rule of 2, characterised by: 2 in. long, 2 feet away from ileocecal valve, occurring in 2% of population, containing 2 types of heterotopic mucosa (gastric and pancreatic), 2 years of age is the most common age of presentation and 2:1 male to female ratio. Commonly, it arises from the anti-mesenteric side of the ileum, located ∼40 cm proximal to the ileocecal valve, containing all 5 layers of small intestine and has a separate mesentery for blood supply [3].

Despite these criteria, the diagnostic concept that a Meckel’s diverticulum must arise from the anti-mesenteric side is challenged by its variant, the mesenteric type that is even rarer, with only few cases reported in the literature. First report was published by Chaffin in 1941, as an unusual variant of Meckel’s diverticulum located on the mesenteric side of the small bowel [3]. Meckel’s diverticulum is either discovered incidentally during surgery for other pathology, or in diagnostic imaging or as a complicated Meckel’s diverticulum. The most common complications include haemorrhage, obstruction, inflammation of the diverticulum, perforation or the presence of a tumour within the diverticulum. Concerns may rise due to the abnormal location of the diverticulum on the mesenteric side as it could rupture or erode into the mesentery putting the patient at risk for bleeding or further serious complications [4].

Haemorrhage, as reported in over 50% of cases, is the most common presentation of Meckel’s diverticulum in young children, while bowel obstruction is the most common in adults. Whereas perforation is considered the least faced complication, as reported in less than 0.5% of symptomatic Meckel’s diverticulum cases [1]. Perforation, however, can be life-threatening and the diagnosis can be delayed or missed considering the overlap of clinical symptoms with other causes of acute abdomen, in addition to the often-non-specific findings on imagining. As previously reported, CT scans and ultrasound are not diagnostic as they are unable to differentiate between a diverticulum and a loop of bowel [5]. Therefore, establishing the correct pre-operative diagnosis can be challenging due to the non-specific symptoms Meckel’s diverticulum can present with. Complicated Meckel’s diverticulum may be often misinterpreted as acute appendicitis with less than 10% of symptomatic cases of Meckel’s diverticulum successfully diagnosed preoperatively [1].

Upon reviewing previous literature and case reports made on similar findings, it was found that they are very often misdiagnosed until the surgical intervention. However, no previous literature reported similar pre-operative diagnosis as our case, where the mesenteric Meckel’s diverticulum was perforated into the inguinal canal, resulting in a sealed perforation leading to the diagnosis of strangulated inguinal hernia. The following is a collection of mesenteric Meckel’s diverticulum cases reported in the surgical literature. The following table, extracted from a previously made table by a previous study [3], alongside 3 extra cases reported recently [1,4,5] list the reported cases in summary (Table 1).

**Table 1:** Summary of mesenteric Meckel’s diverticulum cases reported in the surgical literature.

No.	Author/Yr	Presentation	Pre-op Diagnosis	Procedure	Operative Finding	Histopathology
1	Current Case	70 y/o male with left groin swelling	strangulated inguinal hernia	Segmental bowel resection + side-to-side anastomosis	Perforated mesenteric Meckel’s diverticulum + anti-mesenteric Meckel’s diverticulum	Perforated Meckel’s diverticulum associated with dense inflammation and ischemia
2	Das et al. [11]	32 y/o male with lower abdominal pain	infected collection post umbilical piercing	Small bowel resection + end-to-end anastomosis	Perforated mesenteric Meckel’s diverticulum	Meckel’s diverticulum; gastric mucosa
3	Melissa et al. [4]	27 y/o male with right lower quadrant pain	Acute appendicitis	Small bowel resection + anastomosis	Perforated mesenteric Meckel’s diverticulum	gastric and pancreatic tissue
4	Al-Qahtani et al. [12]	26 y/o female with right lower quadrant pain	Acute appendicitis	Diverticuloectomy	Perforated mesenteric Meckel’s diverticulum	Meckel’s diverticulum with Diverticulitis
5	Abbas et al. [3]	45 y/o female with abdominal pain	Chronic abdominal pain	Segmental bowel resection + end-to-end anastomosis	Mesenteric Meckel’s diverticulum	Mesenteric Meckel’s diverticulum with no malignancy nor ectopic mucosa
6	Toure et al. [16]	45 y/o male with epigastric pain	Acute abdomen	Segmental resection + anastomosis	Perforated mesenteric Meckel’s diverticulum	Perforated diverticulitis without heterotopic tissue
7	Mohanty et al. [13]	16 m/o male with rectal bleeding	Bleeding Meckel’s diverticulum	Small bowel resection + anastomosis	Mesenteric Meckel’s diverticulum	Antral type gastric mucosa
8	Ahmad et al. [2]	25 y/o male with right lower quadrant pain	Small bowel mass	Segmental resection + anastomosis	Mesenteric mass	Chronic inflammation, no heterotopic tissue
9	Singh [7]	2 y/o with umbilical serous discharge 3 y/o with painless rectal bleeding 2 y/o with umbilical pain	--	1 + 2 + 3 = Small bowel resection + anastomosis	1 + 2 + 3 = Mesenteric diverticulum	1=absent gastric mucosa 2 + 3 = Ectopic gastric mucosa
10	Carpenter et al. [14]	35 y/o male with black stool	Meckel’s diverticulum	Small bowel resection + side-to-side anastomosis	Mesenteric diverticulum	Ectopic gastric mucosa
11	Karaman et al. [8]	23 y/o male with right lower quadrant pain		Appendectomy + diverticulectomy	Inflamed Mesenteric diverticulum	Acute appendicitis with Mesenteric diverticulum
12	Seitun et al. [15]	65 y/o female with right lower quadrant pain	Meckel’s diverticulum	Appendectomy + diverticulectomy	Inflammatory changes of distal ileum and diverticulum + mesenteric abscess	Severe acute transmural phlegmonous inflammation; focal area of heterotopic gastric mucosa within the head of the diverticulum with perforation
13	WalczaK et al. [16]	25 y/o male with incidental finding on US	Hypogastric/ mesenteric cyst	Small bowel resection + side-to-side anastomosis	Adherent mesenteric cyst	Ectopic gastric mucosa + inflammatory changes
14	Manukyan et al. [9]	15 y/o female with abdominal pain	Acute abdomen	Ileal segmental resection	Perforated mesenteric diverticulum and pus and intestinal content in pelvis	Pancreatic tissue + oxyntic and antral type gastric mucosa
15	Buke et al. [10]	8 m/o male with Painless rectal bleeding	Meckel’s diverticulum	Segmental resection + anastomosis	Inflamed diverticulum and lymphadenopathy	Heterotopic gastric mucosa
16	Segal et al. [17]	19 y/o male with diffuse abdominal pain	Acute appendicitis	Small bowel resection + wide local excision of mesentery + appendectomy	Mesenteric mass, mesenteric thickening, adenopathy	Ectopic gastric mucosa + inflammatory changes

The table summarises how often, although the total number of cases is few, mesenteric Meckel’s diverticulum cases are misdiagnosed as they present with a variation of non-specific symptoms. However, once intra-operatively encountered, cases of mesenteric Meckel’s diverticulum underwent similar approaches of treatment.

Surgical resection is the treatment of choice for symptomatic Meckel’s diverticulum. Procedures made to achieve treatment most commonly include diverticulectomy, segmental bowel resection and anastomosis and wedge resection [6]. Moreover, finding of an incidental mesenteric Meckel’s should prompt consideration for resection as its location may erode the mesentery and its vasculature causing devastating consequences. Lastly, studies agreed on mesenteric Meckel’s diverticulum resection to avoid further complications [3].

## 4 Conclusion

In summary, Meckel’s diverticulum can be in the mesenteric side despite its classic anti-mesenteric location as a variant, possibly proceeded by a misdiagnosis or a delay of diagnosis until surgery, despite pre-operative workup and examination. Therefore, high clinical suspicion of this pathology and its complications should be encouraged and kept in mind as a differential diagnosis for acute abdomen, as the learning point and purpose of this report, so that timely diagnosis and management of resection could be carried out.

## Declaration of Competing Interest

None of the authors have any conflict of interest to declare.

## Funding

This research did not receive any specific grant from funding agencies in the public, commercial, or not-for-profit sectors.

## Ethical approval

The study is exempt from ethical approval.

## Consent

Signed consent couldn’t be taken from deceased patient nor family members, despite exhaustive attempts made to contact the family. However, the report has been sufficiently anonymised not to cause harm to the patient or their family.

A signed document by the head of department to the above is available.

## Author contribution

Orjuana Khudari gathered patient’s data, designed the case report and drafted the manuscript. Basem AlShareef participated to the manuscript editing to its final version, supervised the report and revised it critically. All authors read and approved the final manuscript to be published.

## Registration of research studies

Not Applicable.

## Guarantor

Orjuana Khudari is the guarantor of submission and accepts full responsibility.

## Provenance and peer review

Not commissioned, externally peer-reviewed.
